# Machine Learning Applied to Proteomic, Metabolomic, and Multi-Omics Biomarkers for the Diagnosis and Risk Stratification of Heart Failure With Preserved Ejection Fraction (HFpEF): A Systematic Review

**DOI:** 10.7759/cureus.106415

**Published:** 2026-04-03

**Authors:** Qossay Alsaafin, Amina Riyaz, Fnu Monishka, Fnu Manesha, Fnu Sandesh, Ahsan Qadeer, Shahbaz Tashfeen

**Affiliations:** 1 Acute Medicine, Royal Albert Edward Infirmary, Wigan, GBR; 2 Cardiology, Moulana Hospital, Perinthalmanna, IND; 3 Internal Medicine, Peoples University of Medical and Health Sciences for Women, Nawabshah, PAK; 4 Internal Medicine, Jinnah Sindh Medical University, Karachi, PAK; 5 Internal Medicine, Mayo Hospital, Lahore, PAK; 6 Internal Medicine, Nishtar Medical University, Multan, PAK

**Keywords:** artificial intelligence, biomarkers, early detection, heart failure with preserved ejection fraction, hfpef, machine learning, metabolomics, multi-omics, proteomics, risk stratification

## Abstract

Heart failure with preserved ejection fraction (HFpEF) is a heterogeneous and increasingly prevalent syndrome that remains challenging to diagnose and risk-stratify using conventional clinical and echocardiographic parameters. Advances in high-throughput proteomic and metabolomic technologies, combined with machine learning methods, have enabled the development of predictive models that capture complex molecular signatures associated with heart failure. This systematic review synthesizes current evidence on machine learning models derived from proteomic, metabolomic, and multi-omics datasets for the diagnosis, early detection, and prognostic assessment of heart failure, with particular focus on HFpEF. A structured search of PubMed, Scopus, and Web of Science identified eight eligible studies published between 2010 and 2026. Included studies applied machine learning techniques to high-dimensional molecular data to predict incident HF, classify HFpEF, identify molecular subtypes, or estimate mortality risk. Several models demonstrated strong discriminatory performance, with reported area under the curve (AUC) or C-index values generally ranging from approximately 0.78 to 0.98, and in some studies, demonstrated improved performance compared with established clinical tools such as natriuretic peptides (e.g., NT-proBNP) and conventional risk scores, including the Meta-Analysis Global Group in Chronic Heart Failure (MAGGIC) score. Multi-omics integration showed particular promise in identifying individuals at risk of developing HFpEF years before symptom onset. However, substantial heterogeneity across molecular platforms, limited external validation in some studies, and vulnerability to overfitting in smaller datasets restrict generalizability. Methodological quality assessment using the Prediction model Risk Of Bias ASsessment tool (PROBAST) tool indicated variable risk of bias, with higher concerns observed in smaller, non-externally validated studies. No randomized trials have yet evaluated the clinical impact of ML-omics-guided risk stratification. Overall, machine learning-based molecular profiling represents a promising direction for refining HFpEF phenotyping and risk prediction, but standardization, cross-platform validation, and outcome-based testing are necessary before routine clinical implementation.

## Introduction and background

Heart failure is a major global health problem and remains one of the leading causes of cardiovascular morbidity and mortality. A substantial proportion of affected individuals present with preserved ejection fraction, commonly referred to as heart failure with preserved ejection fraction [1,2]. Contemporary estimates suggest that HFpEF accounts for approximately 50% or more of all heart failure cases globally, with prevalence increasing across both high-income and low- to middle-income regions due to aging populations and rising cardiometabolic risk factors. This phenotype has become increasingly prevalent and presents important clinical challenges. Diagnosis is often delayed because symptoms are nonspecific and overlap with other cardiopulmonary disorders. Conventional diagnostic tools rely heavily on natriuretic peptides, echocardiography, and clinical assessment, yet these approaches do not fully capture the biological heterogeneity that characterizes this syndrome [3].

There is growing recognition that heart failure, including the preserved ejection fraction subtype, is driven by diverse biological processes involving inflammation, metabolic disturbances, fibrotic pathways, endothelial dysfunction, and alterations in cardiomyocyte signaling [4]. Proteomics and metabolomics have emerged as important strategies to explore these mechanisms through systematic measurement of circulating proteins and metabolites. Key biomarkers implicated in HFpEF pathophysiology include inflammatory mediators such as interleukin-6 and tumor necrosis factor alpha, as well as metabolic markers, including trimethylamine N-oxide and acylcarnitines, which reflect upstream disease processes prior to clinical manifestation [5]. This makes circulating biomarkers attractive candidates for early detection, patient stratification, and risk prediction.

The rapid evolution of machine learning methods has further advanced the study of complex biological data. Techniques such as least absolute shrinkage and selection operator (LASSO), random forest, support vector machines, and gradient boosting algorithms (e.g., XGBoost) enable identification of nonlinear relationships and integration of high-dimensional datasets beyond the capacity of conventional statistical models [6]. These approaches have been applied to classify disease states, predict adverse outcomes, and uncover biological signatures that may improve understanding of disease mechanisms. However, many machine learning models remain limited by reduced interpretability, particularly in more complex or “black-box” algorithms, which may hinder clinical adoption despite strong predictive performance.

Despite these developments, the evidence remains fragmented and heterogeneous. Studies differ in analytic methods, biomarker platforms, validation strategies, clinical endpoints, and population characteristics. There is also a lack of standardization across omics platforms and limited reproducibility of biomarker signatures across cohorts, which complicates comparison and translation into clinical practice. Some studies focus on early diagnosis, while others address risk prediction or longitudinal disease monitoring. Multi-omics integration is increasingly explored, yet the extent to which such approaches improve performance over proteomic or metabolomic models alone remains uncertain [7,8]. In addition, it is important to evaluate whether these models offer advantages over established clinical methods such as natriuretic peptides or traditional risk scores.

Given these considerations, a systematic evaluation of the current evidence is needed. The aim of this review is to synthesize published studies that apply machine learning to proteomic, metabolomic, or multi-omics biomarkers for the diagnosis or risk stratification of heart failure, with particular attention to preserved-ejection-fraction phenotypes. The primary outcome of interest is diagnostic accuracy, while secondary outcomes include prognostic performance and molecular phenotyping. By examining these outcomes alongside model validation and comparative utility, this review seeks to clarify the role of molecular machine learning models in contemporary heart failure assessment and to outline the gaps that should guide future research.

## Review

Materials and methods

Study Design

This systematic review was conducted to synthesize evidence on the application of machine learning (ML) to proteomic, metabolomic, and multi-omics biomarkers for the diagnosis and risk stratification of heart failure (HF), with particular emphasis on heart failure with preserved ejection fraction (HFpEF). The review followed structured methodological principles and incorporated the Population, Intervention, Comparator, and Outcome (PICO) framework to define eligibility criteria [9]. The review process adhered to the Preferred Reporting Items for Systematic Reviews and Meta-Analyses (PRISMA) 2020 guidelines, which are freely available under the Creative Commons Attribution 4.0 International (CC BY 4.0) license, ensuring transparency and reproducibility in study identification, screening, and selection [10]. This review was not prospectively registered in PROSPERO, which represents a methodological limitation. However, a predefined protocol guided the review process, eligibility criteria were clearly specified a priori, and established reporting and methodological frameworks (PRISMA and PICO) were strictly followed to ensure transparency, consistency, and reproducibility. The protocol was developed and applied internally by the authors but was not publicly registered.

Eligibility Criteria (PICO Framework)

The population of interest consisted of adults with established heart failure or individuals at risk of developing HF or HFpEF. The intervention was defined as the use of machine learning models trained on proteomic, metabolomic, or multi-omics datasets, including supervised methods (e.g., logistic regression variants, LASSO), unsupervised approaches (e.g., clustering and similarity-based methods), and, where applicable, more complex algorithms such as tree-based models or ensemble techniques. Comparators included conventional clinical assessments, standard biomarkers, or established risk scores when reported. Eligible outcomes included diagnostic discrimination, prognostic risk prediction, or molecular phenotyping related to heart failure or HFpEF. Studies were included only if they applied machine learning to human proteomic or metabolomic data. Exclusion criteria involved studies based solely on clinical variables, imaging parameters, or genetic data without molecular profiling. Studies limited to genomics-only data were excluded to maintain a focused evaluation of circulating molecular biomarkers that are more directly translatable to clinical practice and comparable across proteomic and metabolomic platforms.

Search Strategy

The literature search was conducted across PubMed, Scopus, and Web of Science to ensure comprehensive coverage of cardiovascular, biomarker, and omics-related publications. Searches spanned January 2010 to January 2026, which reflects the period during which modern high-throughput proteomic and metabolomic platforms became widely available and aligns with the publication range of the included studies. The search strategy used a combination of MeSH terms and free-text keywords linked with Boolean operators. Representative search expressions included “Heart Failure, Diastolic” OR “HFpEF” AND “Proteomics” OR “Metabolomics” OR “Multiomics” AND “Machine Learning” OR “Artificial Intelligence.” Additional expressions included “Heart Failure” AND “SomaScan” OR “Olink” AND “Prediction Model” and “HFpEF” AND “Biomarkers” AND “Logistic Regression” OR “Random Forest” OR “XGBoost.” Titles and abstracts were screened, followed by full-text review according to predefined eligibility criteria.

Study Selection and Data Extraction

Studies were eligible if they used machine learning to analyze proteomic, metabolomic, or multi-omics biomarkers in relation to HF or HFpEF. Once screening was completed, eight studies met the inclusion criteria. Data extraction was performed manually using a standardized data extraction form and included study design, population characteristics, omics platforms, machine learning techniques, clinical objectives, outcomes assessed, model performance metrics, and details of validation. The table of extracted data was developed to ensure consistent formatting and facilitate cross-study comparison.

Quality Assessment

Methodological quality and risk of bias were assessed using the PROBAST tool [11], which is freely available for academic, non-commercial use. PROBAST evaluates four methodological domains: participants, predictors, outcomes, and analysis. Each included study was appraised for risks related to participant selection, biomarker measurement, outcome definition, and modeling strategy. The assessment was conducted independently by two reviewers, and any disagreements were resolved through discussion and consensus, with involvement of a third reviewer when necessary. Studies were subsequently classified as having low, moderate, or high risk of bias, and applicability concerns were documented where relevant. This structured appraisal enabled transparent evaluation of the methodological rigor and reliability of predictive modeling studies included in the review.

Results

Study Selection Process

**Figure 1:**
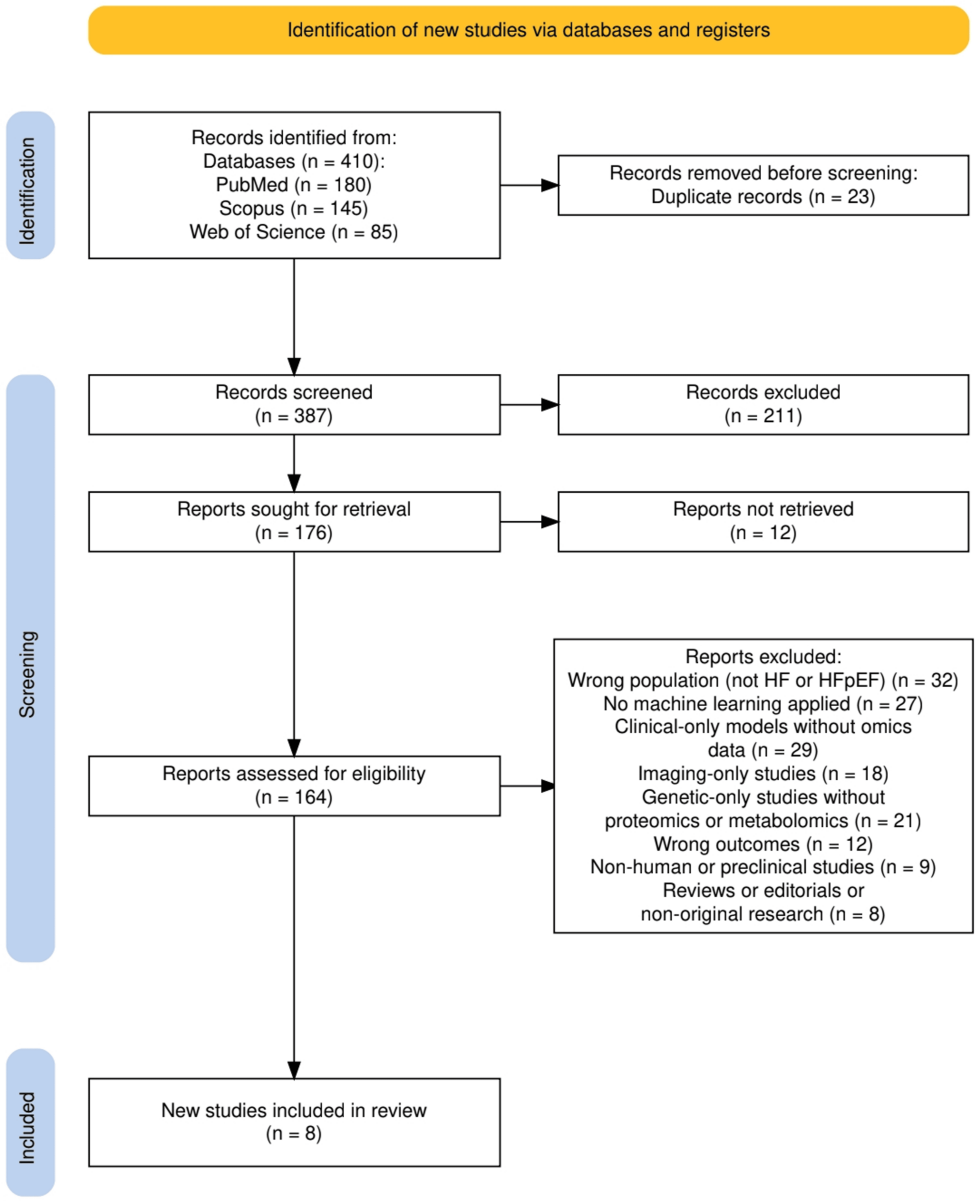
PRISMA flow diagram of the study selection process. This flow diagram is adapted from the PRISMA 2020 guidelines, which are freely available under the Creative Commons Attribution 4.0 International (CC BY 4.0) license [10].

The study selection process followed a structured approach consistent with PRISMA guidelines and is illustrated in Figure 1. A total of 410 records were identified across three databases, including PubMed, Scopus, and Web of Science. After removal of 23 duplicates, 387 records were screened at the title and abstract level, resulting in 211 exclusions. Full texts were sought for 176 studies, of which 12 could not be retrieved. The remaining 164 articles underwent a detailed eligibility assessment based on predefined criteria. A total of 156 studies were excluded for reasons that included wrong population, absence of machine learning methods, omission of proteomic or metabolomic data, reliance on clinical or imaging parameters alone, use of genetic data without molecular profiling, unsuitable outcomes, non-human data, or non-original research formats. Eight studies met all inclusion criteria and were incorporated into the final synthesis.

Characteristics of the Selected Studies

The characteristics of the selected studies are summarized in Table 1, which highlights the diversity of populations, omics platforms, and machine learning (ML) approaches represented in the evidence base. The eight included studies vary in design, ranging from large population cohorts to smaller case-control analyses, and incorporate proteomic, metabolomic, or multi-omics datasets measured through technologies such as SomaScan, Olink, liquid chromatography-mass spectrometry (LC-MS), and targeted metabolite panels. Machine learning methods include logistic regression variants, least absolute shrinkage and selection operator (LASSO), tree-based classifiers, joint models, similarity network fusion (SNF), and multivariable risk score construction. The studies collectively address diagnostic classification, early detection, incident heart failure (HF) prediction, molecular phenotyping, and short-term mortality risk. Model performance across these investigations is consistently strong, although validation strategies differ, with only some studies including large external cohorts. Across modalities, multi-omics models demonstrated the highest overall discriminatory performance, particularly in large cohort-based studies, with reported AUC values exceeding 0.90 in some cases. Proteomic models showed consistently robust and externally validated prognostic performance, whereas metabolomic models demonstrated high diagnostic accuracy in smaller cohorts but were more susceptible to overfitting due to limited sample sizes. The highest performance was observed in multi-omics integration models, such as the study by Versnjak et al., which achieved an AUC ranging from 0.93 to 0.98 for early HFpEF detection. Table 1 provides a clear overview that supports the synthesis of findings presented in this review and illustrates the breadth and methodological heterogeneity of the current machine learning-omics (ML-omics) literature in heart failure with preserved ejection fraction (HFpEF) and HF more broadly.

**Table 1:** Characteristics of the selected studies evaluating machine learning applications in proteomic, metabolomic, and multi-omics biomarkers for heart failure and HFpEF. HF: Heart failure; HFpEF: Heart failure with preserved ejection fraction; HFrEF: Heart failure with reduced ejection fraction; AGES-RS: Age, Gene/Environment Susceptibility–Reykjavik Study; LC-MS: Liquid chromatography–mass spectrometry; LC-MS/MS: Liquid chromatography–tandem mass spectrometry; DIA: Data-independent acquisition; PEA: Proximity Extension Assay; ML: Machine learning; LASSO: Least absolute shrinkage and selection operator; SVM: Support vector machine; XGBoost: Extreme Gradient Boosting; XAI: Explainable artificial intelligence; SNF: Similarity Network Fusion; ROC AUC: Area under the receiver operating characteristic curve; CV: Cardiovascular; LVAD: Left ventricular assist device; MAGGIC: Meta-Analysis Global Group in Chronic Heart Failure; NT-proBNP: N-terminal pro-B-type natriuretic peptide; ELISA: Enzyme-linked immunosorbent assay; C-index: Concordance index.

Study	Design & Population	Omics Type / Platform	ML Method(s)	Clinical Objective	Outcome(s)	Model Performance	Validation
Versnjak et al., 2025 [12]	Prospective UK Biobank cohort; training n=401,917; validation n=100,446; 6,726 HFpEF cases	Multi-omics integration (proteomics, metabolomics, clinical variables)	Supervised classifier; Similarity Network Fusion (SNF)	Early detection and phenotyping of HFpEF	Incident symptom-defined HFpEF; risk subgroup identification	ROC AUC 0.931; Sensitivity 0.857; Specificity 0.847; High-risk cluster AUC 0.988; Detection ~6.3 years before symptoms	Independent hold-out validation subset across 22 centers
Emilsson et al., 2024 [13]	Prospective population cohort (AGES-RS); n=440 incident HF; 188 HFpEF, 167 HFrEF; median follow-up 5.45 years	Proteomics (SomaScan; 4,782 proteins)	LASSO regression with bootstrap	Prediction of incident HF and HF subtypes	Incident HF; HFpEF vs HFrEF subtype prediction	C-index: HF 0.80; HFpEF 0.78; HFrEF 0.80; Strong short-term predictive accuracy (<1 year)	External validation in the Cardiovascular Health Study
de Bakker et al., 2023 [14]	HFrEF cohort; n=382; repeated sampling over median 2.1 years	Proteomics (SomaScan; 4,210 serially measured proteins)	ML-based feature selection + multivariable joint models	Prognostic risk assessment for adverse HF outcomes	Composite endpoint: CV death, transplant, LVAD, HF hospitalization	Cross-validated C-index 0.85; external C-index up to 0.80; outperformed established risk factors	External validation in Henry Ford HF Pharmacogenomic Registry
Baron et al., 2025 [15]	Case-control dataset; n=124 (53 HF, 71 controls)	Metabolomics (targeted panel; 55 metabolites)	Ridge Logistic Regression, SVM, XGBoost; XAI methods (LIME, permutation importance)	Diagnostic classification of HF vs controls	Identification of metabolite signatures predicting HF	Accuracy: 84.0–85.7%; reliable feature importance and interaction insights	Replication cohort validation for key metabolite (C24:0 lignoceric acid)
Kozhevnikova et al., 2025 [16]	Case-control study; n=76 (36 HFpEF, 40 hypertension controls)	Metabolomics (LC-MS; 72 plasma metabolites)	Feature-selected logistic regression model	Diagnostic identification of HFpEF	HFpEF vs hypertension discrimination	AUC 0.981 ± 0.017; statistically significant model (p < 0.001)	Internal validation only
Chadwick et al., 2025 [17]	HFpEF and HFrEF cohorts from 3 studies; training/validation: HFrEF n=1247/762, HFpEF n=725/785; longitudinal subset also included	Proteomics (SomaScan; ~5,000 proteins)	ML-derived proteomic risk scores	Prognostic prediction of 1-year all-cause mortality	HFpEF and HFrEF mortality risk	HFpEF: C-index 0.79, AUC 0.82; Outperformed MAGGIC and NT-proBNP	External validation across multiple independent cohorts
Chen et al., 2026 [18]	Discovery cohort n=36 (12 HFpEF, 12 HFrEF, 12 controls); validation cohort n=180 (60 HFpEF, 60 HFrEF, 60 controls)	Proteomics (Olink PEA Cardiometabolic Panel; 92 proteins)	Logistic regression diagnostic modeling	Identification of HFpEF-specific diagnostic biomarkers	HFpEF vs healthy controls; HFpEF vs HFrEF	AUC > 0.8 for DPP4, KIT, SELL and combined model	Independent ELISA validation cohort
Abudurexiti et al., 2025 [19]	Case-control study; n=40 (20 HFpEF, 20 matched healthy controls)	Proteomics (LC–MS/MS; DIA mode)	LASSO regression; Boruta feature selection	Early diagnostic biomarker discovery for HFpEF	HFpEF vs healthy controls	SERPINA3 AUC 0.835; four candidate biomarkers identified	ELISA validation of selected proteins

Quality Assessment

The quality assessment of the included studies, summarized in Table 2, reveals considerable variation in methodological robustness across the evidence base. Overall, one study was classified as low risk of bias, four as moderate risk, and three as high risk. Studies based on large, well-characterized cohorts with external validation generally demonstrated lower risk of bias, reflecting strong population selection, consistent outcome definitions, and appropriate modeling strategies. In contrast, studies with smaller sample sizes showed a higher risk due to greater susceptibility to overfitting, limited validation, and instability in feature selection when working with high-dimensional omics data. Most studies demonstrated low applicability concerns, although heterogeneity in omics platforms and variation in HFpEF diagnostic definitions introduced moderate concerns in some cases. Table 2 provides a structured comparison of these factors and supports a transparent evaluation of the reliability and generalizability of machine learning omics research in heart failure.

**Table 2:** Quality assessment of included studies based on a PROBAST-informed evaluation. HF: Heart failure; HFpEF: Heart failure with preserved ejection fraction; HFrEF: Heart failure with reduced ejection fraction; ML: Machine learning; DIA: Data-independent acquisition; AUC: Area under the curve; PRS: Proteomic risk score; LASSO: Least absolute shrinkage and selection operator; PROBAST: Prediction Model Risk of Bias Assessment Tool. Risk of bias was assessed using the PROBAST tool, which is freely available for academic use [11].

Study	Participants	Predictors	Outcome	Analysis	Overall Risk of Bias	Applicability Concerns	Notes / Justification
Versnjak et al., 2025 [12]	Low	Low	Low	Moderate	Low–Moderate	Low	Very large biobank cohort; robust phenotyping; advanced multi-omics integration. Analysis complex, some black-box elements and limited interpretability.
Emilsson et al., 2024 [13]	Low	Low	Low	Moderate	Low–Moderate	Low	Strong cohort design, external validation; minor concerns due to LASSO shrinkage and time-to-event modeling not fully detailed.
de Bakker et al., 2023 [14]	Moderate	Low	Low	Moderate	Moderate	Moderate	HFrEF-only limits HFpEF applicability; repeated measures handled with joint models but potential overfitting risk; strong external validation.
Baron et al., 2025 [15]	Moderate	Low	Low	Moderate–High	Moderate–High	Moderate	Small sample size creates overfitting risk; multiple ML models used well, but limited external validation except metabolite replication.
Kozhevnikova et al., 2025 [16]	Moderate	Low	Low	High	High	Moderate	Very small sample, no external validation; simple logistic model; strong AUC likely inflated due to overfitting.
Chadwick et al., 2025 [17]	Low	Low	Low	Low–Moderate	Low	Low	Excellent sample size, multi-cohort validation, rigorous modeling; analysis robust although black-box PRS modeling adds slight concern.
Chen et al., 2026 [18]	Moderate	Low	Low	Moderate	Moderate	Low–Moderate	Good validation cohort; limited sample in discovery; logistic model appropriate but biomarker discovery may inflate performance.
Abudurexiti et al., 2025 [19]	Moderate	Low	Low	High	High	Moderate	Small sample, DIA proteomics good but ML feature selection with no external cohort likely overfits; ELISA validation helps but limited.

Discussion

Integrative Summary

Across the included evidence, machine learning (ML) applied to proteomic, metabolomic, and multi-omics datasets demonstrates clear potential to enhance the diagnostic and prognostic assessment of heart failure (HF). Models trained on molecular biomarkers consistently outperform conventional clinical tools such as natriuretic peptides and established risk scores, suggesting that high-dimensional biochemical information captures disease biology more comprehensively. Heart failure with preserved ejection fraction (HFpEF)-specific models show especially strong discriminatory performance, reflecting the value of molecular profiling in characterizing a syndrome defined by substantial heterogeneity. Multi-omics approaches, such as those presented by Versnjak et al. [12], illustrate that integrated molecular signatures can identify individuals who will develop HFpEF several years before the onset of symptoms. Large proteomic platforms have shown strong prognostic utility for incident HF and mortality in studies such as those by Chadwick et al. [17] and Emilsson et al. [13]. Metabolomic analyses provide complementary biochemical insight and demonstrate reliable discriminative capacity, although sample sizes are often smaller than in proteomic studies. Collectively, the findings indicate that ML models derived from molecular data are reshaping how HFpEF can be detected, phenotyped, and risk-stratified [20,21].

Methodological Strengths and Limitations

The body of evidence shows several methodological strengths, including the use of large population cohorts, multi-cohort validation strategies, and high-dimensional analytic methods that capture the biological diversity of HFpEF. Studies that integrate multiple molecular layers provide important insights into preclinical disease trajectories and reflect the capacity of ML frameworks to detect subtle molecular patterns before clinical manifestation. The use of reproducible proteomic platforms such as SomaScan and Olink also strengthens comparability across studies, as these standardized, high-throughput assays provide consistent and scalable measurement of large numbers of proteins across different cohorts and settings.

However, several limitations must be acknowledged. Smaller metabolomic and proteomic studies, such as those by Kozhevnikova et al. [16], Abudurexiti et al. [19], and Baron et al. [15], face substantial risks of model overfitting due to limited sample size and the high dimensionality of their datasets. Overfitting refers to a model capturing noise or random variation in the training data rather than true underlying patterns, resulting in reduced performance when applied to independent datasets. HFpEF diagnostic criteria vary across studies, which can affect model generalizability. External validation is lacking for several metabolomic models, and the analytical structure of some ML approaches limits interpretability, raising concerns about clinical usability. Importantly, none of the studies incorporate prospective clinical trial testing to determine whether these models improve patient outcomes [22]. The predominance of data from high-income countries also limits global applicability. These methodological considerations are essential for contextualizing the promise of ML-omics approaches in HFpEF.

Absence of Cross-Platform or Cross-Cohort Biomarkers

A notable gap identified in this review is the absence of consistent biomarker replication across different proteomic and metabolomic platforms. Each study employed distinct technologies, including SomaScan in the proteomic investigations by Emilsson et al. [13] and Chadwick et al. [17], LC-MS-based metabolomics in the work of Baron et al. [15] and Kozhevnikova et al. [16], and Olink assays in the study by Chen et al. [18]. This diversity in measurement techniques limits direct comparison of biomarker signatures and restricts the ability to identify candidates that demonstrate stability across analytic systems. For example, proteins such as DPP4, KIT, and SELL identified in Olink-based analyses [18] were not consistently replicated in SomaScan-based proteomic studies, while metabolite signatures such as lignoceric acid (C24:0) identified in targeted metabolomic models [15] were not observed across other cohorts or platforms. As a result, proteins and metabolites that perform well in one study seldom appear in others, which prevents the establishment of a universal HFpEF molecular signature. This fragmentation represents a critical barrier to clinical translation because reproducibility across cohorts, platforms, and molecular modalities is fundamental for the development of reliable diagnostic or prognostic tools [23]. Addressing this gap will require harmonized biomarker panels, standardized data processing pipelines, and coordinated efforts to validate molecular signatures across independent populations.

HFpEF as a Multi-Domain Molecular Disease

The findings across the reviewed studies support a broader conceptualization of HFpEF as a multi-domain molecular disorder rather than a condition confined to cardiac dysfunction. Inflammatory pathways are prominent, demonstrated by changes in molecules such as SELL, KIT, and cytokine-related proteins described in the proteomic analyses of Chen et al. [18] and Emilsson et al. [13]. Cardiometabolic dysregulation also emerges as a central theme, reflected in signals involving DPP4, ADMA, TMAO, and various carnitine species that were highlighted in the studies of Kozhevnikova et al. [16] and Baron et al. [15]. Additionally, extracellular matrix remodeling appears relevant, with SERPINA3 and ITIH4 identified by Abudurexiti et al. [19].

This constellation of biological disturbances suggests that HFpEF involves interconnected processes spanning metabolic, inflammatory, and structural domains across multiple organ systems. Multi-omics machine learning frameworks, such as those used by Versnjak et al. [12], capture these diverse abnormalities more effectively than traditional clinical markers, which typically focus on limited physiological parameters. This integrated perspective reinforces the idea that HFpEF is a systemic condition that requires biologically informed diagnostic strategies.

Translational Potential and Barriers to Clinical Adoption

The growing body of machine learning omics research shows considerable translational promise, as these models offer non-invasive avenues for risk prediction, earlier diagnosis, and improved stratification for clinical trials. Molecular profiling can identify biologically distinct subtypes of HFpEF, which is particularly relevant for a condition marked by heterogeneity and limited therapeutic options. Subtype identification also provides a framework for individualized treatment approaches and more targeted enrollment in intervention studies. Despite these advantages, several barriers limit immediate clinical adoption [24]. Access to advanced omics platforms remains uneven, and the associated costs challenge widespread implementation, particularly in low- and middle-income settings where resource constraints may limit availability of high-throughput proteomic and metabolomic technologies. Standardization across assays and platforms is still lacking, which complicates comparison and reproducibility. Regulatory frameworks for machine learning models in cardiovascular medicine are evolving and require robust evidence for safety and reliability. Integration of these tools into electronic health records will require substantial workflow modifications and informatics resources. Clinician confidence in the interpretability and transparency of machine learning models is also necessary to support routine use [25]. These obstacles underscore the need for coordinated research, infrastructural development, and policy guidance before omics-based models can be incorporated into clinical care.

Contribution of This Review to the Literature

This review contributes several important insights to the existing literature on molecular machine learning models for heart failure, with specific relevance to HFpEF. It offers the first integrated synthesis of proteomic, metabolomic, and multi-omics evidence that applies machine learning techniques to both diagnostic and prognostic questions in HFpEF and related heart failure subtypes. Comparing molecular models with established clinical markers, this analysis highlights consistent gains in discriminatory performance across multiple platforms and study designs. The structured assessment of methodological quality using PROBAST [11] allows for a transparent evaluation of risk of bias and underscores the limitations of smaller studies that are more vulnerable to overfitting. The review identifies multi-omics integration as a promising approach for detecting HFpEF at early stages and for revealing molecular subtypes that may inform personalized care. It also draws attention to platform heterogeneity as a major impediment to reproducibility and emphasizes the need for harmonized analytical frameworks. Together, these contributions provide a clearer understanding of the current state of the field and outline the methodological and translational challenges that must be addressed going forward [26,27].

Evidence Gaps and Future Research Directions

Several important evidence gaps emerge from the current body of machine learning omics research in HFpEF. Most available studies rely on single-center or homogeneous cohorts, and there is a lack of large, multi-ethnic datasets that would allow robust assessment across diverse populations and comorbidity patterns. Prognostic research specific to HFpEF remains limited, with most studies focusing on diagnostic discrimination rather than longitudinal risk prediction. Replication of biomarker signatures across different proteomic and metabolomic platforms is uncommon, which restricts generalizability and prevents the identification of reliable cross-cohort molecular markers. No randomized trials have yet evaluated whether ML-omics stratification improves clinical outcomes, and longitudinal multi-omics datasets remain scarce, limiting the ability to characterize disease progression in molecular terms. Future work should prioritize harmonized definitions of HFpEF, standardized molecular assays, and coordinated multi-platform validation. There is a clear need for larger studies that incorporate diverse populations and that test multi-omics deep learning models with transparent and interpretable components. Integration of ML-omics tools into clinical trial enrichment strategies may help identify biologically defined HFpEF subgroups and improve study efficiency. Ultimately, future research must evaluate the effect of ML-omics-guided decision-making on patient outcomes to determine the clinical value of these approaches.

## Conclusions

The evidence reviewed in this study demonstrates that machine learning models built on proteomic, metabolomic, and multi-omics data offer meaningful advances in the detection and characterization of heart failure, particularly HFpEF. These models capture complex molecular patterns that are not accessible through traditional clinical assessment or single biomarkers, and several studies show that they can identify disease risk or subtypes with strong discriminatory performance. Although the translational potential is considerable, important limitations persist, including variability in analytic platforms, limited external validation for some models, and the absence of outcome-based trials. Addressing these gaps will require coordinated, large-scale, and methodologically rigorous research that evaluates molecular signatures across diverse populations and integrates these tools into clinical workflows. The take-home message is that ML-omics approaches have the capacity to transform HFpEF diagnosis and risk stratification, but their clinical impact will depend on standardization, reproducibility, and evidence that they improve patient care.
